# From reservoirs to ecological integrators: the role of European *Apodemus* spp. in vector-borne zoonotic pathogens

**DOI:** 10.3389/fvets.2026.1822333

**Published:** 2026-06-04

**Authors:** Daniele Fabbri, Luca Dorigo, Laura Grassi, Stefania Leopardi, Valentina Tagliapietra, Paola Beraldo

**Affiliations:** 1 Department of Agricultural, Food, Environmental and Animal Sciences, University of Udine, Udine, Italy; 2 NBFC, National Biodiversity Future Center, Palermo, Italy; 3 Museo Friulano di Storia Naturale, Udine, Italy; 4 Istituto Zooprofilattico Sperimentale delle Venezie, Campoformido, Italy; 5 Istituto Zooprofilattico Sperimentale delle Venezie, Legnaro, Italy; 6 Research and Innovation Center, Fondazione Edmund Mach, San Michele all’Adige, Italy

**Keywords:** *Apodemus*, host-vector ecology, One Health, reservoir competence, vector-borne and zoonotic diseases

## Abstract

Rodents of the genus *Apodemus* are among the most widespread and abundant small mammals in Europe and play a central role in the ecology of numerous zoonotic pathogens. Owing to their ecological plasticity, high population densities, and frequent infestation by arthropod vectors, *Apodemus* species contribute to the maintenance and transmission of a diverse array of bacterial, viral, and protozoan agents of public health concern. This narrative review synthesises current knowledge on vector-borne pathogens associated with European *Apodemus* spp. and highlights marked heterogeneity in species-specific epidemiological roles across pathogens. We highlight marked heterogeneity in the epidemiological roles of *Apodemus* spp. across pathogens. While they function as confirmed reservoir hosts for some agents, particularly certain *Borrelia* genospecies and *Neoehrlichia mikurensis*, their contribution to other transmission cycles is ecotype-dependent, indirect, or context-specific. Infection dynamics are shaped by host demography, population density, community composition, habitat configuration, and climatic variability, which together modulate vector abundance, host-vector contact rates, and environmental persistence. Importantly, infection prevalence alone does not equate to reservoir competence; instead, pathogen-specific life histories and ecological context determine the extent to which *Apodemus* spp. amplify or bridge transmission. Landscape structure, forest cover, and temperature-related variables emerge as key large-scale predictors of tick-borne pathogen incidence, linking rodent ecology to human disease risk. Urbanisation and land-use change further reorganise host–vector networks, creating novel interfaces for spillover. By integrating multi-pathogen evidence across ecological scales, this review underscores the importance of adopting a One Health perspective that considers rodent ecology, vector biology, and environmental change in concert. Understanding the context-dependent role of *Apodemus* spp. is essential for anticipating future zoonotic risk under ongoing climatic and landscape transformation.

## Graphical abstract

**Figure:**
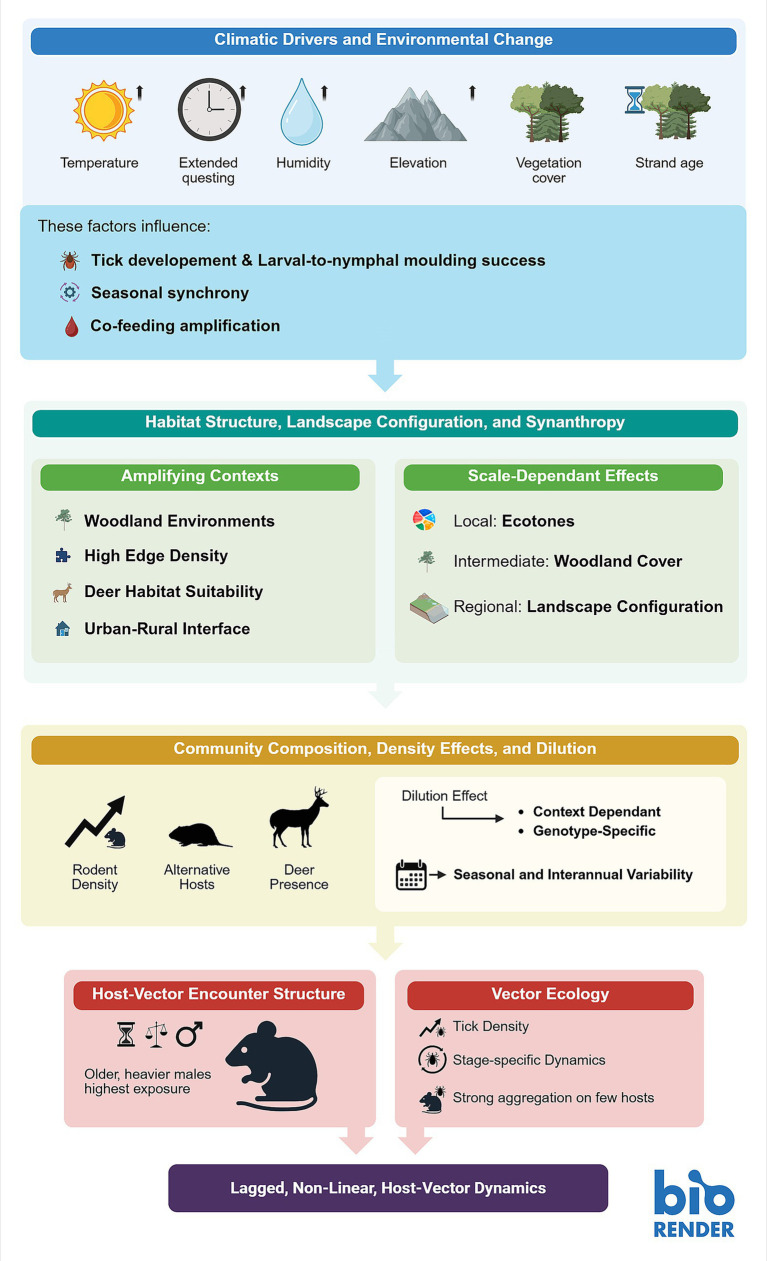
Multi-scale ecological determinants of zoonotic pathogen transmission in *Apodemus* spp. Transmission intensity (infected nymph density) emerges from hierarchical ecological filtering in which climatic drivers shape habitat suitability and tick phenology, landscape configuration structures host–vector encounters, and multi-host community dynamics modulate amplification or dilution. Vector ecology, particularly tick density, stage synchrony, aggregation on few hosts, and lagged host–vector dynamics, exerts a stronger influence on transmission than host demography alone. Effects are nonlinear, scale-dependent, and context specific, such that changes in rodent density or climate do not necessarily translate into proportional increases in infected nymph density.

## Introduction

Vector-borne zoonoses are increasingly recognised as a major public health challenge in Europe, driven by shifts in climate, land use, and wildlife community composition (1–3). Arthropod vectors such as ticks, fleas, and mites depend on small mammal hosts to complete their life cycles, thereby linking wildlife reservoirs with human exposure (1, 2). Among these hosts, rodents are particularly influential due to their high abundance, rapid demographic turnover, and capacity to sustain substantial ectoparasite burdens.

Rodents are ubiquitous components of terrestrial ecosystems and occur across forested, agricultural, and peri-urban landscapes throughout Europe. Through seed and fungal spore dispersal (4, 5), pollination, and their role as prey for mesocarnivores and raptors (6–9), they contribute to trophic connectivity and ecosystem functioning. Their widespread distribution and high local abundance also position them as recurrent hosts within arthropod life cycles, supporting immature stages of ticks, fleas, and mites and thereby structuring the ecological networks through which pathogens circulate. Despite these functions, wild rodents are often regarded primarily as agricultural pests due to crop damage and contamination (10, 11), a perception that can obscure their broader ecological and epidemiological importance.

Within European small mammal communities, several taxonomic groups contribute to vector-borne transmission cycles, including murid mice (e.g., *Apodemus* and *Mus* spp.), arvicoline voles (e.g., *Myodes*, *Microtus*, and *Arvicola* spp.), and soricid shrews (e.g., *Crocidura* and *Sorex* spp.). Among these, species of the genus *Apodemus* are particularly prominent due to their broad geographic distribution, high local abundance, and frequent infestation by immature stages of arthropod vectors. They occur across Mediterranean, Eastern, and Central European regions, occupying a wide range of forested, agricultural, and peri-urban habitats where vector-host interactions are intensified.

Several *Apodemus* species are recognised across Europe, including *A. agrarius* and members of the *Sylvaemus* group: *A. alpicola, A. flavicollis, A. mystacinus, A. sylvaticus, A. uralensis,* and *A. witherbyi* (12, 13). Although *A. ponticus* has occasionally been treated as a distinct species, it is more commonly considered conspecific with *A. flavicollis*. The close morphological similarity and overlapping distributions of these taxa often complicate field identification (14). Such taxonomic complexity has important implications for epidemiological studies, as misidentification may obscure species-specific patterns of vector infestation and pathogen prevalence.

Small mammals function as reservoir and amplification hosts within arthropod-mediated transmission networks, maintaining pathogens that circulate between vectors and vertebrate communities (3). In these systems, pathogen persistence depends not only on host competence but also on the frequency and intensity of ectoparasite infestation. Ticks, fleas, and other hematophagous arthropods acquire infections during blood meals and subsequently transmit them to new hosts, thereby structuring enzootic cycles and shaping human exposure risk (1, 2).

Within European small mammal communities that sustain these transmission cycles, Apodemus species occupy a central position among small mammal hosts in arthropod-mediated transmission systems. They are among the most frequent hosts of immature stages of *Ixodes ricinus*, as well as fleas (e.g., *Ctenophthalmus* spp.) and gamasid mites (15, 16). Repeated vector feeding on these mice facilitates the acquisition and onward transmission of pathogens such as tick-borne encephalitis virus (TBEV), *Borrelia burgdorferi sensu lato*, *Anaplasma phagocytophilum*, *Rickettsia* spp., and *Bartonella* spp. High local densities, combined with substantial ectoparasite burdens, enable Apodemus populations to function as efficient amplification hosts within enzootic cycles, sustaining pathogen circulation across seasons (17).

Beyond their central role in vector feeding networks, demographic characteristics of *Apodemus* populations further modulate vector-borne transmission dynamics. High reproductive rates and rapid cohort turnover generate continuous pools of susceptible hosts, sustaining pathogen circulation over time. Their short lifespans (typically 6–18 months) and multiannual fluctuations (18, 19), driven by resource availability and predation pressure, influence seasonal patterns of vector infestation and infection prevalence (20). Age- and sex-related differences also shape transmission: older individuals often carry heavier ectoparasite burdens, and males frequently exhibit higher infection rates, potentially reflecting increased movement, aggressive interactions, and greater exposure to questing vectors (21).

Climate change is expected to reshape the epidemiology of arthropod-borne pathogens across Europe by modifying vector phenology, survival, and geographic distribution. Temperature and precipitation regimes influence the development, activity periods, and overwintering success of ticks and other ectoparasites, thereby altering transmission windows and host–vector encounter rates. Within this context, fluctuations in *Apodemus* populations can further amplify climate-driven effects. In the Mediterranean basin, for example, increased autumn and winter precipitation has been associated with population growth of the wood mouse (*A. sylvaticus*), with higher rainfall linked to increased reproductive success (22) and greater capture rates (23), while *A. flavicollis* similarly exhibits abundance peaks following wet years (24).

Extreme precipitation events and associated flooding can further intensify vector-borne transmission by modifying both habitat structure and pathogen dispersal pathways. Floodwaters may redistribute infected ectoparasites and increase contact rates at the wildlife-human interface, while simultaneously facilitating environmental contamination of surface waters (25, 26). In parallel, climate-driven shifts in vegetation phenology, such as increasingly frequent mast seeding in deciduous forests, can elevate host densities and thereby enhance blood-meal availability for ticks, contributing to population growth of *Ixodes ricinus* (27). Such cascading effects are expected to influence the spatial and temporal dynamics of tick-borne encephalitis, with incidence projected to increase both in established endemic regions and toward the northern limits of its distribution (28).

When superimposed on projected socioeconomic transitions and land-use change, these climate-mediated shifts in vector and host dynamics may disproportionately increase exposure risk in lower-income countries and vulnerable communities (29). Uneven access to surveillance infrastructure, healthcare systems, and vector control measures can further amplify the public health consequences of expanding arthropod-borne pathogens. Addressing these multifactorial risks requires an integrated One Health framework that jointly considers vector ecology, small mammal population dynamics, environmental change, and human behavioural factors to strengthen early detection and mitigation strategies.

This review examines the role of Apodemus species within European arthropod-borne pathogen systems, focusing on bacterial and viral agents maintained through vector-mediated transmission. Rather than addressing rodent-borne zoonoses broadly, it specifically evaluates the evidence supporting their function as reservoir and amplification hosts across multiple vector taxa. By integrating insights from vector ecology, small mammal demography, and climate-driven environmental change, this synthesis seeks to clarify transmission mechanisms and inform targeted surveillance, risk assessment, and prevention strategies for vector-borne diseases associated with *Apodemus* spp.

## Methods

### Search criteria

This narrative review was developed through targeted literature retrieval conducted between February and October 2025. The scope was restricted to peer-reviewed primary studies published in English between 2000 and 2025 that reported original empirical evidence of vector-borne zoonotic pathogen detection, occurrence, or prevalence in European populations of *Apodemus* spp.

Europe was defined according to the United Nations Statistics Division (UNSD) M49 regional classification, excluding territories of the Russian Federation located east of the Ural Mountains.

This geographic framework was applied consistently during database filtering and study selection. Bibliographic searches were conducted in PubMed, Web of Science, Google Scholar, and the CABI Digital Library. Search strings were constructed by combining host terms, pathogen-specific terms, and geographic identifiers with Boolean operators, using syntax adapted to each database. The full database-specific search queries are provided in Supplementary Table S1.

Exclusion criteria comprised reviews, non-European studies, non-vector-borne pathogens, studies without original host-level data, and papers in which *Apodemus* occurrence could not be linked to pathogen detection. Screening was performed in two stages: initial evaluation of titles and abstracts followed by full-text assessment when relevance was uncertain. Duplicate entries were identified and removed prior to qualitative synthesis. Studies reporting *Apodemus* only at genus level were retained when species-level identification was unavailable, but these records were interpreted cautiously because taxonomic uncertainty may obscure species-specific epidemiological patterns.

For each eligible study, information on pathogen identity, vector associations, host species, geographic location, diagnostic method, and reported prevalence was recorded. When available, ecological factors potentially influencing transmission dynamics, such as host density, demographic structure, habitat characteristics, and climatic conditions, were extracted to contextualise epidemiological patterns. Given the heterogeneity of study designs and the limited quantitative comparability across pathogens, findings were synthesised narratively rather than subjected to formal meta-analytical procedures.

## Results

### Study selection and overview of included evidence

Studies included in this review were identified through structured literature retrieval across PubMed, Web of Science, Google Scholar, and the CABI Digital Library, followed by relevance screening of titles, abstracts, and, where necessary, full texts. Because the review was designed as a narrative synthesis rather than a formal systematic review, study selection did not follow a fully standardised quantitative screening workflow. The synthesis was organised at the level of included studies rather than database hits, and studies reporting *Apodemus* only at genus level were retained when species-level identification was unavailable.

The final corpus covered a broad geographic range across Europe and included evidence on viral, bacterial, and protozoan pathogens associated with *Apodemus* spp. Most of the reviewed literature concerned tick-borne bacteria, particularly *Borrelia* spp., *Anaplasma phagocytophilum*, *Neoehrlichia mikurensis*, and *Rickettsia* spp., whereas evidence for some pathogens, such as West Nile virus and Crimean–Congo haemorrhagic fever virus, was much more limited. Across the included studies, *A. flavicollis*, *A. sylvaticus*, and *A. agrarius* were the most frequently represented host taxa, while records for *A. uralensis* and genus-level *Apodemus* spp. were less common. Diagnostic approaches were dominated by polymerase chain reaction (PCR)-based methods, including reverse-transcription polymerase chain reaction (RT-PCR), with additional evidence derived from enzyme-linked immunosorbent assay (ELISA), immunofluorescence assay (IFA), including indirect immunofluorescence assay (IIFA), and microscopy-based methods. Within the latter category, blood smear examination was recorded separately when studies explicitly reported stained blood films, whereas broader microscopy-based analyses were retained for studies reporting microscopic examination without sufficient methodological detail to classify the preparation more precisely.

### Pathogens reported in European *Apodemus* spp. with stronger evidence of epidemiological involvement

#### Viral pathogens

##### Flaviviridae

Tick-borne encephalitis virus (TBEV) was reported in 10 studies from eight European countries, whereas West Nile virus (WNV) was represented by a single study from Italy (Table 1). Reported host taxa included *A. agrarius*, *A. flavicollis*, *A. sylvaticus*, and records identified only as *Apodemus* spp., with evidence generated mainly by RT-PCR, ELISA, IFA, and IIFA.

**Table 1:** Detection of Flaviviridae (tick-borne encephalitis virus [TBEV] and West Nile virus [WNV]) in European *Apodemus* populations.

Host species	Pathogen(s)	*n*/*N*	(%)	Method	Country	References
*A. agrarius*	TBEV	ns	–	RT-PCR	Estonia	(121)
*Apodemus* spp.	TBEV	11/625	1.8	ELISA	France	(122)
*Apodemus* spp.	TBEV	10/280	3.6	ELISA	France	(92)
*A. agrarius*	TBEV	3/24	12.5	RT-PCR	Germany	(73)
*A. flavicollis*	TBEV	10/123	8.1	RT-PCR	Germany	(73)
*A. sylvaticus*	TBEV	2/7	28.6	RT-PCR	Germany	(73)
*A. flavicollis*	TBEV	19/181	10.5	IIFA	Germany	(98)
*A. agrarius*	TBEV	2/25	8.0	RT-PCR	Hungary	(123)
*A. flavicollis*	TBEV	4/100	4.0	RT-PCR	Hungary	(123)
*A. flavicollis*	TBEV	12/327	3.7	ns	Hungary	(124)
*A. agrarius*	TBEV	8/174	4.6	ns	Hungary	(124)
*A. flavicollis*	WNV	4/60	6.7	ELISA	Italy	(79)
*A. agrarius*	TBEV	1/1	100	RT-PCR	Lithuania	(125)
*A. flavicollis*	TBEV	59/76	77.6	RT-PCR	Lithuania	(125)
*A. sylvaticus*	TBEV	2/2	100	RT-PCR	Lithuania	(125)
*A. sylvaticus*	TBEV	3/113	2.7	RT-PCR	Netherlands	(74)
*A. agrarius*	TBEV	4/160	2.5	IFA	Slovenia	(126)
*A. flavicollis*	TBEV	33/820	4.0	IFA	Slovenia	(126)
*A. sylvaticus*	TBEV	7/66	10.6	IFA	Slovenia	(126)
*A. flavicollis*	TBEV	1/77	1.3	IFA	Switzerland	(127)
*A. sylvaticus*	TBEV	3/104	2.9	IFA	Switzerland	(127)

The table summarises country, host species, diagnostic method, and number of positive individuals over total tested as reported in each study. ns, not specified.

Detection frequencies varied markedly among studies, ranging from isolated positives to high focal proportions, including 1/77 in Switzerland, 3/24 in Germany, 12/327 in Hungary, 33/820 in Slovenia, and 59/76 in Lithuania. Species-level detections were most frequently reported for *A. flavicollis* and *A. agrarius*, although several studies presented pooled *Apodemus* records without species resolution.

West Nile virus (WNV) was represented by a single study from Italy, in which antigens were detected in *A. flavicollis* by ELISA (4/60). Compared with TBEV, evidence for WNV in European *Apodemus* populations was therefore sparse and geographically limited within the reviewed literature.

#### Bacterial pathogens

##### *Borrelia* spp.

*Borrelia* spp. were the most frequently reported pathogens in the reviewed literature, with detections documented across a broad set of European studies from 18 countries: Austria, Croatia, Czechia, France, Germany, Hungary, Italy, Lithuania, the Netherlands, Norway, Poland, Portugal, Romania, Serbia, Slovakia, Slovenia, Spain, and Switzerland (Table 2). Reported host taxa included *A. agrarius*, *A. flavicollis*, *A. sylvaticus*, *A. uralensis*, and records identified only as *Apodemus* spp., with evidence generated mainly by PCR-based methods and, less frequently, by ELISA.

**Table 2:** Detection of *Borrelia* spp. in European *Apodemus* populations.

Host species	Pathogen(s)	*n*/*N*	(%)	Method	Country	References
*A. agrarius*	*B. burgdorferi s.l.*	1/32	3.1	PCR	Austria	(128)
*A. flavicollis*	*B. afzelii, B. burgdorferi s.s., B. garinii*	100/414	24.2	PCR	Austria	(129)
*A. sylvaticus*	*B. afzelii, B. burgdorferi s.s., B. burgdorferi s.l., B. garinii*	51/66	77.3	PCR	Austria	(129)
*A. flavicollis*	*B. afzelii*	3/29	10.3	RT-PCR	Austria	(130)
*A. sylvaticus*	*B. afzelii*	1/26	3.8	RT-PCR	Austria	(130)
*A. agrarius*	*B. afzelii, B. miyamotoi*	8/53	15.1	PCR	Croatia	(131)
*A. flavicollis*	*B. afzelii, B. miyamotoi*	3/131	2.3	PCR	Croatia	(131)
*A. agrarius*	*B. burgdorferi s.l.*	8/32	25.0	PCR	Czechia	(132)
*Apodemus* spp.	*B. burgdorferi s.l.*	27/297	9.1	PCR	Czechia	(132)
*A. flavicollis*	*B. burgdorferi s.l.*	3/37	8.1	ELISA	Czechia	(133)
*A. sylvaticus*	*B. burgdorferi s.l.*	2/10	20.0	ELISA	Czechia	(133)
*A. flavicollis*	*B. burgdorferi s.l.*	25/168	14.9	ELISA	Czechia	(134)
*A. sylvaticus*	*B. burgdorferi s.l.*	nr	–	PCR	France	(135)
*A. sylvaticus*	*B. burgdorferi s.l.*	14/450	3.1	PCR	France	(102, 106)
*A. flavicollis*	*B. afzelii*	2/8	25.0	PCR	France	(136)
*A. sylvaticus*	*B. afzelii*	5/11	45.5	PCR	France	(136)
*A. flavicollis*	*B. afzelii*	28/59	47.5	PCR	Germany	(137)
*A. agrarius*	*B. afzelii*	1/90	1.1	PCR	Germany	(138)
*A. flavicollis*	*B. afzelii*	4/240	1.7	PCR	Germany	(138)
*A. sylvaticus*	*B. afzelii*	2/108	1.9	PCR	Germany	(138)
*A. agrarius*	*B. afzelii*	3/7	42.9	PCR	Germany	(139)
*A. flavicollis*	*B. afzelii, B. burgdorferi s.l.*	64/214	29.9	PCR	Germany	(139)
*A. flavicollis*	*B. burgdorferi s.l., B. miyamotoi*	6/102	5.9	PCR	Hungary	(40)
*A. agrarius*	*B. burgdorferi s.l.*	16/202	7.9	PCR	Hungary	(40)
*Apodemus* spp.	*B. afzelii, B. lusitaniae, B. valaisiana*	6/81	7.4	PCR	Italy	(140)
*Apodemus* spp.	*B. burgdorferi s.l.*	7/101	6.9	PCR	Italy	(141)
*A. agrarius*	*B. afzelii, B. burgdorferi s.l.*	2/30	6.7	PCR	Lithuania	(142)
*A. flavicollis*	*B. afzelii, B. burgdorferi s.l.*	9/85	10.6	PCR	Lithuania	(142)
*A. flavicollis*	*B. burgdorferi s.l., B. miyamotoi*	6/29	20.7	PCR	Netherlands	(108)
*A. sylvaticus*	*B. afzelii, B. burgdorferi s.l., B. miyamotoi*	34/373	9.1	PCR	Netherlands	(108)
*A. sylvaticus*	*B. afzelii*	22/128	17.2	PCR	Netherlands	(100)
*A. sylvaticus*	*B. burgdorferi s.l., B. miyamotoi*	47/338 approx.	13.9	PCR	Netherlands	(93)
*A. sylvaticus*	*B. burgdorferi s.l.*	82/281 approx.	29.2	PCR	Norway	(96)
*A. sylvaticus*	*B. afzelii, B. burgdorferi s.l.*	33/172	19.2	PCR	Norway	(143)
*A. flavicollis*	*B. burgdorferi s.l.*	1/19	5.3	PCR	Norway	(143)
*A. agrarius*	*B. afzelii, B. burgdorferi s.l.*	2/41	4.9	PCR	Norway	(142)
*A. flavicollis*	*B. afzelii, B. burgdorferi s.l.*	6/101	5.9	PCR	Norway	(142)
*A. agrarius*	*B. afzelii, B. miyamotoi*	12/157	7.6	PCR	Poland	(41, 109)
*A. flavicollis*	*B. afzelii, B. miyamotoi*	5/84	6.0	PCR	Poland	(41, 109)
*A. flavicollis*	*B. burgdorferi s.l.*	4/163	2.5	PCR	Poland	(144)
*A. sylvaticus*	*B. lusitaniae*	1/22	4.5	PCR	Portugal	(145)
*A. agrarius*	*B. afzelii, B. garinii, B. burgdorferi s.s.*	3/87	3.4	PCR	Romania	(146)
*A. flavicollis*	*B. afzelii, B. miyamotoi*	1/49	2.0	PCR	Romania	(146)
*A. flavicollis*	*B. burgdorferi s.l.*	2/104	1.9	PCR	Serbia	(147)
*A. agrarius*	*B. miyamotoi*	1/48	2.1	PCR	Serbia	(147)
*A. flavicollis*	*B. afzelii, B. bavariensis, B. garinii, B. miyamotoi*	33/356	9.3	PCR	Slovakia	(97)
*A. sylvaticus*	*B. afzelii*	1/52	1.9	PCR	Slovakia	(97)
*A. flavicollis*	*B. afzelii, B. garinii*	22/76	28.9	PCR	Slovakia	(148)
*A. flavicollis*	*B. burgdorferi s.l.*	38/182	20.9	PCR	Slovakia	(103)
*A. agrarius*	*B. burgdorferi s.l.*	19/97	19.6	PCR	Slovakia	(103)
*A. uralensis*	*B. burgdorferi s.l.*	1/15	6.7	PCR	Slovakia	(103)
*A. agrarius*	*B. burgdorferi s.l.*	9/45	20.0	ELISA	Slovakia	(149)
*A. flavicollis*	*B. burgdorferi s.l.*	29/135	21.5	ELISA	Slovakia	(149)
*A. uralensis*	*B. burgdorferi s.l.*	2/45	4.4	ELISA	Slovakia	(149)
*A. agrarius*	*B. afzelii*	2/12	16.7	PCR	Slovakia	(150)
*A. flavicollis*	*B. afzelii*	9/60	15.0	PCR	Slovakia	(150)
*A. flavicollis*	*B. burgdorferi s.l.*	8/19	42.1	ELISA	Slovakia	(151)
*A. agrarius*	*B. burgdorferi s.l.*	3/30	10.0	ELISA	Slovakia	(151)
*A. flavicollis*	*B. afzelii, B. miyamotoi*	4/18	22.2	PCR	Slovenia	(152)
*A. sylvaticus*	*Borrelia* spp. *strain R57*	52/162	32.1	PCR	Spain	(153)
*A. sylvaticus*	*B. burgdorferi s.l.*	3/36	8.3	PCR	Spain	(154)
*A. sylvaticus*	*B. burgdorferi s.l., B. burgdorferi s.s.*	16/130	12.3	PCR	Spain	(155)
*A. flavicollis*	*B. afzelii, B. garinii*	nr	–	PCR	Switzerland	(156)
*A. sylvaticus*	*B. afzelii, B. garinii*	1/6	16.7	PCR	Switzerland	(156)

The table summarises host species, *Borrelia* genospecies detected, country, diagnostic method, and number of positive individuals over total tested, with studies organised alphabetically by country and first author. nr, not reported.

Detection frequencies varied widely among studies, from isolated positives to high proportions in focal populations. Examples include 1/32 in *A. agrarius* from Austria, 28/59 in *A. flavicollis* from Germany, 33/356 in *A. flavicollis* from Slovakia, 52/162 in *A. sylvaticus* from Spain, 82/281 in *A. sylvaticus* from Norway, and 100/414 in *A. flavicollis* from Austria. Across the reviewed studies, *A. flavicollis* and *A. sylvaticus* were the most frequently represented hosts, although several studies also reported pooled *Apodemus* records without species-level resolution.

The most frequently reported genospecies was *B. afzelii*, often detected alone or together with *B. burgdorferi sensu lato*, whereas *B. miyamotoi* was reported in a smaller subset of studies. Additional taxa reported less frequently included *B. garinii*, *B. burgdorferi* sensu stricto, *B. lusitaniae*, *B. valaisiana*, *B. bavariensis*, and Borrelia spp. strain R57. Co-detections involving *B. afzelii* and *B. miyamotoi* were reported in several studies from Central and Eastern Europe.

##### Ehrlichia muris

*Ehrlichia muris* was reported in a small number of studies from four European countries: Croatia, Poland, Slovakia, and Ukraine (Table 3). Reported host taxa included *A. flavicollis* and *A. agrarius*, and detections were obtained mainly by PCR, with one additional report based on microscopical analysis.

**Table 3:** Detection of *Ehrlichia muris* in European *Apodemus* populations.

Host species	Pathogen(s)	*n*/*N*	(%)	Method	Country	References
*A. flavicollis*	*Ehrlichia* spp.	1/131	0.8	PCR	Croatia	(131)
*A. agrarius*	*Ehrlichia* spp.	1/53	1.9	PCR	Croatia	(131)
*A. agrarius*	*Ehrlichia* spp.	5/14	35.7	PCR	Poland	(157)
*A. flavicollis*	*Ehrlichia* spp.	1/19	5.3	PCR	Slovakia	(157)
*A. flavicollis*	*Ehrlichia* spp.	1/19	5.3	PCR	Slovakia	(158)
*A. flavicollis*	*Ehrlichia* spp.	2/15	13.3	Microscopic analysis	Ukraine	(159)

The table summarises host species, country, diagnostic method, and number of positive individuals over total tested, with studies organised alphabetically by country and first author.

Detection frequencies were generally low, including 1/131 in *A. flavicollis* and 1/53 in *A. agrarius* from Croatia, 1/19 in *A. flavicollis* from Slovakia, and 2/15 in *A. flavicollis* from Ukraine, although one Polish study reported 5/14 positives in *A. agrarius*. Overall, reports in *Apodemus* populations were sparse and geographically limited within the reviewed literature.

##### Neoehrlichia mikurensis

*Neoehrlichia mikurensis* was reported in European *Apodemus* populations in studies from nine countries: Austria, Czechia, France, Germany, Hungary, the Netherlands, Slovakia, Sweden, and Switzerland (Table 4). Reported host taxa included *A. agrarius*, *A. flavicollis*, *A. sylvaticus*, and records identified only as *Apodemus* spp., with detections generated almost exclusively by PCR-based methods, including one RT-PCR study from Austria.

**Table 4:** Detection of *Neoehrlichia mikurensis* in European *Apodemus* populations.

Host species	Pathogen(s)	*n*/*N*	(%)	Method	Country	References
*A. agrarius*	*N. mikurensis*	2/32	6.3	RT-PCR	Austria	(128)
*A. flavicollis*	*N. mikurensis*	6/48	12.5	PCR	Czechia	(160)
*A. sylvaticus*	*N. mikurensis*	2/6	33.3	PCR	Czechia	(160)
*A. sylvaticus*	*N. mikurensis*	2/79	2.5	PCR	France	(161)
*A. flavicollis*	*N. mikurensis*	35/59	59.3	PCR	Germany	(137)
*A. agrarius*	*N. mikurensis*	4/78	5.1	PCR	Germany	(162)
*A. flavicollis*	*N. mikurensis*	10/82	12.2	PCR	Germany	(162)
*A. flavicollis*	*N. mikurensis*	50/178	28.1	PCR	Germany	(64)
*A. sylvaticus*	*N. mikurensis*	1/36	2.8	PCR	Germany	(64)
*A. agrarius*	*N. mikurensis*	1/3	33.3	PCR	Germany	(45)
*A. flavicollis*	*N. mikurensis*	24/37	64.9	PCR	Germany	(45)
*A. agrarius*	*N. mikurensis*	3/92	3.3	PCR	Hungary	(163)
*A. flavicollis*	*N. mikurensis*	3/67	4.5	PCR	Hungary	(163)
*A. flavicollis*	*N. mikurensis*	7/29	24.1	PCR	Netherlands	(108)
*A. sylvaticus*	*N. mikurensis*	50/370	13.5	PCR	Netherlands	(108)
*A. sylvaticus*	*N. mikurensis*	5/23	21.7	PCR	Netherlands	(46)
*A. agrarius*	*N. mikurensis*	1/324	0.3	PCR	Slovakia	(47)
*A. flavicollis*	*N. mikurensis*	6/181	3.3	PCR	Slovakia	(47)
*Apodemus* spp.	*N. mikurensis*	22/358	6.1	PCR	Slovakia	(164)
*Apodemus* spp.	*N. mikurensis*	nr	–	PCR	Slovakia	(165)
*Apodemus* spp.	*N. mikurensis*	2/42	4.8	PCR	Sweden	(166)
*A. flavicollis*	*N. mikurensis*	1/45	2.2	PCR	Switzerland	(48)
*A. sylvaticus*	*N. mikurensis*	4/35	11.4	PCR	Switzerland	(48)

The table summarises host species, country, diagnostic method, and number of positive individuals over total tested, with studies organised alphabetically by country and first author. nr, not reported.

Detection frequencies ranged from low to comparatively high across studies. Examples included 2/79 in *A. sylvaticus* from France, 6/48 in *A. flavicollis* from Czechia, 7/29 in *A. flavicollis* and 50/370 in *A. sylvaticus* from the Netherlands, 24/37 in *A. flavicollis* from Germany, and 50/178 in *A. flavicollis* in another German study. Lower-frequency detections were also reported in *A. agrarius*, including 2/32 in Austria, 3/92 in Hungary, and 1/324 in Slovakia.

Across the reviewed literature, *A. flavicollis* and *A. sylvaticus* accounted for most positive reports, although genus-level *Apodemus* records were also present in Slovakia and Sweden. Overall, the evidence base indicated a broad geographic distribution and repeated detection of *N. mikurensis* in multiple *Apodemus* taxa across Central and Western Europe.

###### *Rickettsia* spp.

*Rickettsia* spp. were reported in *Apodemus* populations from 11 European countries: Austria, Croatia, Czechia, Germany, Italy, Lithuania, the Netherlands, Poland, Serbia, Slovakia, and Ukraine (Table 5). Reported host taxa included *A. agrarius*, *A. flavicollis*, *A. sylvaticus*, *A. uralensis*, and records identified only as *Apodemus* spp., with detections generated mainly by PCR or RT-PCR, together with a small number of IFA and microscopical reports.

**Table 5:** Detection of *Rickettsia* spp. in European *Apodemus* populations.

Host species	Pathogen(s)	*n*/*N*	(%)	Method	Country	References
*A. flavicollis*	ns	2/29	6.9	RT-PCR	Austria	(130)
*A. sylvaticus*	ns	2/26	7.7	RT-PCR	Austria	(130)
*A. flavicollis*	ns	2/115	1.7	RT-PCR	Croatia	(167)
*A. agrarius*	ns	2/32	6.3	PCR	Czechia	(132)
*Apodemus* spp.	ns	21/297	7.1	PCR	Czechia	(132)
*A. agrarius*	ns	8/90	8.9	PCR	Germany	(167)
*A. flavicollis*	*R. helvetica*	37/240	15.4	PCR	Germany	(168)
*A. sylvaticus*	Not specified	20/108	18.5	PCR	Germany	(168)
*A. agrarius*	*R. helvetica*	4/45	8.9	PCR	Germany	(65)
*A. flavicollis*	*R. helvetica, R. felis, R. raoultii*	130/925	14.1	PCR	Germany	(65)
*A. flavicollis*	*R. helvetica*	16/59	27.1	PCR	Germany	(137)
*A. agrarius*	*R. helvetica*	2/3	66.7	PCR	Germany	(66)
*A. flavicollis*	*R. helvetica*	14/37	37.8	PCR	Germany	(66)
*A. sylvaticus*	*R. helvetica*	17/105	16.2	PCR	Germany	(66)
*A. flavicollis*	*R. helvetica, R. felis*	50/214	23.4	PCR	Germany	(139)
*A. flavicollis*	*R. conorii, R. helvetica*	4/19	21.1	IFA	Germany	(169)
*A. sylvaticus*	*R. conorii, R. helvetica*	4/6	66.7	IFA	Germany	(169)
*Apodemus* spp.	*R. slovaca*	8/37	21.6	PCR	Italy	(67)
*Apodemus* spp.	*R. slovaca*	6/81	7.4	PCR	Italy	(140)
*A. flavicollis*	*R. helvetica*	68/231	29.4	PCR	Lithuania	(170)
*A. flavicollis*	ns	2/29	6.9	PCR	Netherlands	(108)
*A. sylvaticus*	*R. helvetica*	38/374	10.2	PCR	Netherlands	(108)
*Apodemus* spp.	*R. conorii, R.* sp. *IRS, R. helvetica*	65/146	44.5	PCR	Netherlands	(171)
*A. flavicollis*	*R. felis*	5/25	20.0	PCR	Poland	(172)
*A. agrarius*	*R. helvetica*	25/96	26.0	PCR	Poland	(172)
*A. agrarius*	Not specified	1/48	2.1	PCR	Serbia	(147)
*A. flavicollis*	*R. helvetica, R. monacensis*	5/104	4.8	PCR	Serbia	(147)
*A. agrarius*	*R. helvetica*	1/69	1.4	PCR	Slovakia	(173)
*A. flavicollis*	*R. helvetica, R. felis, R. slovaca*	10/99	10.1	PCR	Slovakia	(173)
*A. uralensis*	ns	1/1	100	PCR	Slovakia	(173)
*A. agrarius*	*R. helvetica*	1/8	12.5	PCR	Slovakia	(174)
*A. flavicollis*	*R. helvetica*	14/247	5.7	PCR	Slovakia	(174)
*A. agrarius*	*R. helvetica*	20/340	5.9	PCR	Slovakia	(175)
*A. flavicollis*	*R. helvetica*	12/312	3.8	PCR	Slovakia	(175)
*A. flavicollis*	*R. helvetica*	3/45	6.7	PCR	Slovakia	(42)
*A. flavicollis*	ns	7/15	46.7	Microscopic analysis	Ukraine	(159)
*A. sylvaticus*	ns	1/6	16.7	Microscopic analysis	Ukraine	(159)

The table summarises host species, *Rickettsia* species detected, country, diagnostic method, and number of positive individuals over total tested, with studies organised alphabetically by country and first author. ns, not specified.

Detection frequencies varied substantially among studies, from isolated positives to relatively high proportions in focal populations. Examples included 2/29 in *A. flavicollis* from Austria, 37/240 in *A. flavicollis* from Germany, 68/231 in *A. flavicollis* from Lithuania, 65/146 in *Apodemus* spp. from the Netherlands, 25/96 in *A. agrarius* from Poland, and 20/340 in *A. agrarius* from Slovakia. Across the reviewed literature, *A. flavicollis* was the most frequently represented host, although positive records were also reported in *A. agrarius*, *A. sylvaticus*, *A. uralensis*, and genus-level *Apodemus* records.

The most frequently reported species was *R. helvetica*, which was detected across multiple countries and host taxa. Other reported taxa included *R. felis*, *R. slovaca*, *R. conorii*, *R. monacensis*, *R. raoultii*, and one record reported as *Rickettsia* sp. IRS, whereas several studies did not resolve detections beyond *Rickettsia* spp. or reported unspecified positives. Overall, the evidence indicated broad but taxonomically uneven detection of *Rickettsia* in European *Apodemus* populations, with *R. helvetica* dominating the dataset.

#### Parasitic pathogens

##### Babesia microti

*Babesia* spp., predominantly *B. microti*, were reported in European *Apodemus* populations from 12 countries: Bulgaria, Croatia, Czechia, Germany, Ireland, Lithuania, the Netherlands, Poland, Serbia, Slovakia, Slovenia, and Ukraine (Table 6). Reported host taxa included *A. agrarius*, *A. flavicollis*, *A. sylvaticus*, and records identified only as *Apodemus* spp., with detections generated mainly by PCR, together with a smaller number of blood-smear and microscopical reports.

**Table 6:** Detection of *Babesia* spp. in European *Apodemus* populations.

Host species	Pathogen(s)	*n*/*N*	(%)	Method	Country	References
*A. agrarius*	*B. microti*	1/31	3.2	Blood smears	Bulgaria	(176)
*A. flavicollis*	*B. microti*	6/37	16.2	PCR	Croatia	(54)
*A. flavicollis*	ns	5/28	17.9	PCR	Croatia	(177)
*A. agrarius*	*B. microti*	3/53	5.7	PCR	Croatia	(131)
*A. flavicollis*	*B. microti*	4/131	3.1	PCR	Croatia	(131)
*A. agrarius*	ns	1/32	3.1	PCR	Czechia	(132)
*Apodemus* spp.	ns	15/297	5.1	PCR	Czechia	(132)
*A. flavicollis*	*B. microti*	1/178	0.6	PCR	Germany	(55)
*A. agrarius*	*B. microti*	1/3	33.3	PCR	Germany	(119)
*A. sylvaticus*	*B. microti*	5/314	1.6	PCR	Ireland	(178)
*A. agrarius*	ns	1/48	2.1	PCR	Lithuania	(179)
*A. flavicollis*	*B. microti*	11/499	2.2	PCR	Lithuania	(180)
*A. sylvaticus*	*B. microti*	6/374	1.6	PCR	Netherlands	(108)
*A. flavicollis*	*B. microti*	1/85	1.2	PCR	Poland	(181)
*A. flavicollis*	ns	1/104	1.0	PCR	Serbia	(147)
*A. agrarius*	*B. microti*	12/324	3.7	PCR	Slovakia	(47)
*A. flavicollis*	*B. microti*	4/350	1.1	PCR	Slovakia	(47)
*Apodemus* spp.	*B. microti*	7/195	3.6	PCR	Slovakia	(182)
*A. agrarius*	*B. microti*	3/370	0.8	Blood smears	Slovakia	(183)
*A. agrarius*	*B. microti*, *Babesia* spp.	3/60	5.0	PCR	Slovakia	(150)
*A. flavicollis*	*B. microti*	15/127	11.8	PCR	Slovenia	(184)
*A. flavicollis*	ns	10/15	66.7	Microscopic analysis	Ukraine	(159)
*A. sylvaticus*	ns	3/6	50.0	Microscopic analysis	Ukraine	(159)

The table summarises host species, country, diagnostic method, and number of positive individuals over total tested, with studies organised alphabetically by country and first author. ns, not specified.

Detection frequencies ranged from isolated positives to relatively high proportions in some local studies. Examples included 1/178 in *A. flavicollis* from Germany, 5/314 in *A. sylvaticus* from Ireland, 6/374 in *A. sylvaticus* from the Netherlands, 12/324 in *A. agrarius* from Slovakia, 15/127 in *A. flavicollis* from Slovenia, and 10/15 in *A. flavicollis* from Ukraine. Across the reviewed literature, both *A. agrarius* and *A. flavicollis* were repeatedly represented, although several studies also reported genus-level *Apodemus* records without species resolution.

Most studies that resolved the pathogen identified *B. microti*, whereas a smaller number reported unspecified *Babesia* spp. or mixed entries such as *B. microti* / *Babesia* spp. The reviewed evidence therefore indicated repeated detection of *B. microti* across multiple *Apodemus* taxa and countries, with occasional records lacking species-level parasite identification.

### Pathogens reported in European *Apodemus* spp. with limited or context-dependent evidence

#### Viral pathogens

##### Orthonairovirus haemorrhagiae

Crimean–Congo haemorrhagic fever virus (CCHFV; *Orthonairovirus haemorrhagiae*) was represented by a single study from Hungary in the reviewed literature (Table 7). In that study, 18 of 1,439 *A. flavicollis* individuals were positive, as well as 1 of 42 *A. agrarius.* No additional European studies reporting CCHFV detection in *Apodemus* populations were identified in the reviewed dataset, indicating that evidence for this pathogen in European *Apodemus* hosts remains very limited.

**Table 7:** Detection of *Orthonairovirus* (CCHF) in European *Apodemus* populations.

Host species	*n*/*N*	(%)	Method	Country	References
*A. flavicollis*	18/1439	1.3	IFA	Hungary	(120)
*A. agrarius*	1/42	2.4	IFA	Hungary	(120)

The table reports host species, country, diagnostic method, and number of positive individuals over total tested as described in the referenced studies.

#### Bacterial pathogens

##### Anaplasma phagocytophilum

*Anaplasma phagocytophilum* was reported in European *Apodemus* populations from at least 13 countries or country groupings: Belgium/Netherlands, Bulgaria, Czechia, France, Germany, Italy, the Netherlands, Romania, Serbia, Slovakia, Spain, Switzerland, and the United Kingdom (Table 8). Reported host taxa included *A. agrarius*, *A. flavicollis*, *A. sylvaticus*, *A. uralensis*, and records identified only as *Apodemus* spp., with detections generated predominantly by PCR and, in a smaller number of cases, by ELISA.

**Table 8:** Detection of *Anaplasma phagocytophilum* in European *Apodemus* populations.

Host species	*n*/*N*	(%)	Method	Country	References
*A. sylvaticus*	1/23	4.3	PCR	Belgium & Netherlands	(185)
*A. agrarius*	3/9	33.3	PCR	Bulgaria	(186)
*A. agrarius*	2/32	6.2	PCR	Czechia	(132)
*Apodemus* spp.	26/297	8.8	PCR	Czechia	(132)
*A. flavicollis*	6/40	15.0	PCR	Czechia	(187)
*A. flavicollis*	11/54	20.4	PCR	France	(58)
*A. sylvaticus*	2/6	33.3	PCR	France	(58)
*A. sylvaticus*	2/18	11.1	PCR	France	(188)
*A. sylvaticus*	20/452	4.4	PCR	France	(102, 106)
*A. flavicollis*	1/218	0.5	PCR	Germany	(189)
*A. flavicollis*	4/82	4.9	PCR	Germany	(162)
*A. agrarius*	1/78	1.3	PCR	Germany	(162)
*A. sylvaticus*	2/25	8.0	PCR	Germany	(162)
*A. flavicollis*	1/178	0.6	PCR	Germany	(64)
*A. sylvaticus*	2/36	5.6	PCR	Germany	(64)
*A. flavicollis*	3/685	0.4	PCR	Italy	(59)
*A. sylvaticus*	2/198	1.0	PCR	Netherlands	(108)
*A. flavicollis*	5/77	6.5	PCR	Romania	(60)
*A. sylvaticus*	2/51	3.9	PCR	Romania	(60)
*A. uralensis*	3/62	4.8	PCR	Romania	(60)
*A. flavicollis*	1/104	1.0	PCR	Serbia	(147)
*A. agrarius*	1/296	0.3	PCR	Slovakia	(190)
*A. flavicollis*	2/239	0.8	PCR	Slovakia	(190)
*Apodemus* spp.	nr	–	PCR	Slovakia	(165)
*A. flavicollis*	2/60	3.3	PCR	Slovakia	(150)
*A. flavicollis*	2/38	5.3	PCR	Slovakia	(191)
*A. flavicollis*	nr	–	PCR	Slovakia	(69)
*A. agrarius*	7/45	15.6	ELISA	Slovakia	(149)
*A. flavicollis*	23/135	17.0	ELISA	Slovakia	(149)
*A. uralensis*	2/45	4.4	ELISA	Slovakia	(149)
*A. sylvaticus*	1/162	0.6	PCR	Spain	(153)
*A. flavicollis*	2/69	2.9	PCR	Switzerland	(192)
*A. sylvaticus*	7/390	1.8	PCR	United Kingdom	(193)

The table summarises host species, country, diagnostic method, and number of positive individuals over total tested as reported in the referenced studies. nr, not reported.

Detection frequencies varied markedly across studies, from isolated positives to higher focal proportions. Examples included 1/23 in *A. sylvaticus* from Belgium/Netherlands, 3/9 in *A. agrarius* from Bulgaria, 11/54 in *A. flavicollis* from France, 5/77 in *A. flavicollis* from Romania, 7/45 in *A. agrarius* and 23/135 in *A. flavicollis* in one Slovak study, and 7/390 in *A. sylvaticus* from the United Kingdom. Several studies also reported low-frequency detections in Germany, Italy, Serbia, Spain, and Switzerland.

Across the reviewed literature, *A. flavicollis* and *A. sylvaticus* were the most frequently represented hosts, although positive records were also reported in *A. agrarius*, *A. uralensis*, and genus-level *Apodemus* records. The evidence base therefore indicated broad but heterogeneous detection of *A. phagocytophilum* in European *Apodemus* populations, with substantial variation among studies in both host representation and reported positivity.

##### Coxiella burnetii

*Coxiella burnetii* was reported in European *Apodemus* populations from four countries: Czechia, Italy, Slovakia, and Spain (Table 9). Reported host taxa included *A. agrarius*, *A. flavicollis*, *A. sylvaticus*, and records identified only as *Apodemus* spp., with detections generated by both PCR and ELISA.

**Table 9:** Detection of *Coxiella burnetii* in European *Apodemus* populations.

Host species	*n*/*N*	(%)	Method	Country	References
*A. agrarius*	7/40	17.5	PCR	Czechia	(194)
*A. flavicollis*	30/324	9.3	PCR	Czechia	(194)
*A. sylvaticus*	2/48	4.2	PCR	Czechia	(194)
*A. flavicollis*	6/37	16.2	ELISA	Czechia	(133)
*A. sylvaticus*	4/10	40.0	ELISA	Czechia	(133)
*A. flavicollis*	34/168	20.2	ELISA	Czechia	(134)
*Apodemus* spp.	2/101	2.0	PCR	Italy	(141)
*A. flavicollis*	1/38	2.6	PCR	Slovakia	(191)
*A. flavicollis*	nr	–	PCR	Slovakia	(69)
*A. sylvaticus*	1/162	0.6	PCR	Spain	(153)
*A. sylvaticus*	12/138	8.7	PCR	Spain	(195)

The table summarises host species, country, diagnostic method, and number of positive individuals over total tested as reported in each study. nr, not reported.

Detection frequencies varied across studies. In Czechia, positives were reported in *A. agrarius* (7/40), *A. flavicollis* (30/324 and 34/168), and *A. sylvaticus* (2/48 and 4/10). Additional PCR-based detections were reported in *Apodemus* spp. from Italy (2/101), *A. flavicollis* from Slovakia (1/38, with one additional unspecified positive record), and *A. sylvaticus* from Spain (1/162 and 12/138).

## Pathogen-specific interpretation

The reviewed evidence indicates that *Apodemus* spp. occupy heterogeneous epidemiological roles across vector-borne pathogen systems rather than a uniform reservoir status, with patterns ranging from confirmed or strongly inferred rodent-associated maintenance to context-dependent or incidental involvement depending on pathogen life history and transmission ecology (30–34).

At the strongest end of this gradient, *Borrelia* spp., especially *B. afzelii*, and *Neoehrlichia mikurensis* show the most consistent evidence of close association with *Apodemus* hosts. The enzootic maintenance of *Borrelia burgdorferi sensu lato* depends on reservoir-competent vertebrates that infect feeding larvae and nymphs, with *Ixodes ricinus* acting as the principal European vector and transovarial transmission considered negligible (35–37). Within this system, *Apodemus* spp. function both as frequent hosts of immature ticks and as important reservoirs for *B. afzelii*, promoting the production of infected nymphs that drive human Lyme borreliosis risk (31, 38, 39). A similarly strong interpretation applies to *B. miyamotoi*, for which *Apodemus* spp. are considered primary reservoir hosts in Europe, even though other mammals and birds may also contribute locally (40–44). *Neoehrlichia mikurensis* also lies near the reservoir-competence end of the gradient. In Europe, its primary vector is *I. ricinus*, and rodents, including *Apodemus* spp., are widely regarded as the main reservoir hosts sustaining enzootic transmission (34, 45–49).

Certain *Babesia* spp., particularly *B. microti*, also fall toward the stronger end of host involvement, although the evidence is somewhat less uniform than for *Borrelia* or *N. mikurensis*. *B. microti* is primarily associated with small rodents, is transmitted by *Ixodes* ticks, and has confirmed zoonotic potential (50, 51). Unlike several bacterial tick-borne pathogens, *Babesia* spp. can also be transmitted transovarially, allowing persistence within tick populations independent of continuous vertebrate amplification (52, 53). Even so, wild rodents, including *Apodemus* spp., are widely regarded as key reservoirs sustaining local transmission cycles, although genetic variation among *B. microti* isolates suggests that zoonotic significance may differ among lineages (54–56).

By contrast, the role of *Anaplasma phagocytophilum* is more clearly ecotype-dependent and geographically variable. Its epidemiology is structured into ecotypes with distinct host associations and zoonotic potential, with rodent-associated ecotypes circulating mainly within small mammal–tick systems and generally considered non-zoonotic (57). Rodents are clearly involved in local maintenance, but the inferred role of *Apodemus* spp. varies among studies, with some suggesting greater competence in arvicoline rodents such as *Myodes glareolus*, and others reporting comparable or higher prevalence in *Apodemus* populations (58–63). This pattern is more consistent with evidence of host participation and context-dependent amplification than with a uniform reservoir role (57, 64).

The evidence for *Rickettsia* spp. and *Ehrlichia muris* is weaker and should be interpreted more cautiously. *Rickettsia* spp. are repeatedly detected in *Apodemus* tissues, and *Apodemus* hosts often carry ticks with high *Rickettsia* prevalence (65–67). However, rickettsial maintenance also relies heavily on efficient vertical transmission in ticks, which complicates inference about vertebrate reservoir importance (68). For *E. muris*, detections in *Apodemus* remain rare and geographically limited, while tick-based evidence suggests broader circulation than rodent data alone would indicate (69–72). For both pathogen groups, current evidence supports detection and possible host involvement, but not a strong claim of confirmed reservoir competence in European *Apodemus* spp. (66, 67, 69, 70).

At the weakest end of the gradient, flaviviruses, *Coxiella burnetii*, and Crimean–Congo haemorrhagic fever virus appear to involve *Apodemus* more indirectly or under restricted ecological conditions. For TBEV, small rodents are clearly involved in local foci because they host immature ticks and facilitate co-feeding transmission, even when systemic viraemia is limited (73–76). However, the epidemiological effect of *Apodemus* on TBEV transmission is tightly constrained by vector synchrony, co-feeding intensity, and broader host–vector context (77, 78). For WNV, the case is weaker still: birds are the principal amplifying hosts, whereas mammals are generally considered dead-end or low-competence hosts, and evidence for *Apodemus* involvement remains limited (79–83). Small rodents also do not appear central to CCHFV maintenance, which depends primarily on *Hyalomma* ticks and larger vertebrate amplification hosts (30, 84–87). Similarly, although rodents may participate in local wildlife cycles of *C. burnetii*, their importance appears secondary to domestic ruminants, which remain the principal reservoirs and major source of human infection (32, 88–91).

Overall, pathogen detections in *Apodemus* should not be treated as interchangeable evidence of reservoir competence. Rather, the reviewed literature supports a gradient from strong rodent-associated maintenance in *Borrelia* spp. and *N. mikurensis*, through ecotype- or context-dependent roles in *A. phagocytophilum* and *Babesia* spp., to more uncertain, indirect, or incidental involvement for *Rickettsia* spp., *E. muris*, *WNV*, *C. burnetii*, and CCHFV (30–32, 34, 55, 57, 69).

## Ecological determinants of infection of zoonotic pathogens in *Apodemus* spp.

### Vector life history and phenology

In vector-borne systems, transmission intensity is primarily structured by vector ecology rather than host demography alone. The seasonal activity, developmental synchrony, and host-seeking behaviour of *Ixodes* ticks determine when and how frequently *Apodemus* individuals are exposed to infection. For tick-borne encephalitis virus (TBEV), rodent abundance indirectly influences viral amplification because small mammals serve as primary hosts for immature ticks, facilitating co-feeding transmission within local foci (75). However, the prevalence of TBEV in questing nymphs may vary seasonally without proportional changes in infected nymph density (92), underscoring the importance of stage-specific vector dynamics.

Longitudinal evidence further indicates that large fluctuations in rodent density do not necessarily translate into equivalent changes in infected nymph density, reflecting lagged host-vector dynamics, strong aggregation of ticks on particular hosts, and high larval-to-nymphal moulting success (76, 93, 94). Similarly, modelling approaches in north-western Europe have shown that variability in Lyme borreliosis risk is explained primarily by tick density rather than by shifts in the proportion of putative dilution hosts, with only a low probability of a strong dilution effect under those ecological conditions (95).

### Host-vector encounter structure

Demographic correlates of infection frequently reflect differential exposure to feeding ticks rather than intrinsic susceptibility. Tick burdens commonly increase with host body mass, and male rodents may carry disproportionately higher larval and nymphal loads (94, 96, 97). Older individuals are more frequently infected with flaviviruses such as TBEV, plausibly due to cumulative exposure and repeated tick encounters (98, 99).

However, sex bias and age effects are not universally consistent across years or species, indicating that demographic patterns are context dependent and mediated by ecological variability (93, 98). Importantly, tick infestation is highly aggregated: a minority of rodents may feed a disproportionate fraction of larvae, thereby contributing disproportionately to the pool of infected nymphs (93). Thus, apparent demographic effects often reflect heterogeneity in vector feeding intensity rather than uniform differences in host competence.

### Community composition, density effects, and dilution

Transmission unfolds within multi-host assemblages in which community composition strongly modulates pathogen amplification. Spatial variation in Lyme borreliosis risk depends not only on rodent infection prevalence but also on alternative vertebrate hosts and vector aggregation patterns (100). Although the dilution effect hypothesis predicts declining nymphal infection prevalence with increasing host diversity, European evidence suggests a more nuanced pattern (101). Dilution effects may not operate uniformly and may depend on genospecies-specific dynamics.

For *Borrelia* spp., prevalence in *Apodemus* populations often increases toward autumn and exhibits marked interannual variability (97, 102, 103). Although tick abundance does not consistently predict individual infection probability (102), it frequently explains more variation in overall disease risk than host diversity metrics alone (95). At the same time, in heterogeneous agricultural mosaics, increasing overall small-mammal community abundance has been associated with reduced tick prevalence and lower mean tick burdens in dominant rodent hosts, consistent with dilution effects under specific ecological conditions (15).

### Habitat structure, landscape configuration, and synanthropy

Habitat composition and landscape structure shape infection risk by modulating vector survival and host–vector contact. Woodland environments generally sustain higher immature tick densities than open habitats, particularly where woody vegetation maintains favourable microclimatic conditions for questing (15). Forested landscapes with high edge density tend to amplify transmission by increasing encounter probability between hosts and ticks, whereas at the scale of individual forest patches, greater ecotone availability may exert diluting effects (104, 105). At broader spatial scales, deer habitat suitability and vegetation productivity further link landscape configuration to spatial variation in Lyme borreliosis incidence (105), while increasing land-use intensity in heterogeneous mosaics can reduce tick prevalence under specific conditions (15).

Pathogen responses to habitat structure are similarly scale dependent. *Anaplasma phagocytophilum* prevalence increases with wooded habitat cover at intermediate spatial scales, likely via host population size, whereas *Borrelia burgdorferi sensu lato* prevalence responds primarily to wooded ecotones at local scales (106). Multi-regional analyses of deciduous forest fragments further demonstrate that habitat properties may exert stronger effects on *B. burgdorferi* prevalence than macroclimate or landscape configuration alone (104).

Urbanisation and synanthropic dynamics add another layer of complexity. Transitional urban–rural interfaces can concentrate exposure to tick-borne pathogens (107), and urban green spaces may sustain *Borrelia* circulation in *Apodemus* populations (108–110). Simplified urban mammal assemblages often retain a subset of zoonotic agents that persist or increase despite reduced biodiversity (111).

### Climatic drivers and environmental change

Climatic variability shapes tick development, seasonal synchrony, and habitat suitability, thereby influencing pathogen transmission. Temperature-related variables are among the strongest predictors of tick density and human TBEV occurrence across Europe (112, 113). Warming scenarios project increased transmission potential through extended questing seasons, larger susceptible larval and nymphal cohorts, and enhanced co-feeding opportunities that amplify non-systemic transmission (114). Consistent with this, TBEV-positive *Ixodes ricinus* have been detected at elevations above former distribution limits in the Czech Republic following significant regional warming (115).

Moisture conditions also contribute to pathogen maintenance: relative humidity correlates strongly with TBEV prevalence in Scandinavia, suggesting a role in sustaining viral circulation in emerging foci (33). Forest cover, particularly mature high-stand forest, further promotes TBEV persistence by enhancing habitat suitability for both ticks and small mammal hosts (75, 112, 116, 117).

However, climatic effects are not uniformly amplifying. Climate-driven increases in rodent density or tick activity do not necessarily translate into proportional increases in infected nymph density, owing to buffering by tick aggregation and high moulting success (93). In many systems, habitat quality and landscape configuration explain more variation in *Borrelia* prevalence than macroclimate alone (104), and land-use complexity can modulate or dampen climate-associated risk (15, 118). These nonlinear, scale-dependent interactions underscore the need for long-term ecological surveillance rather than short-term prevalence snapshots.

### Integrative ecological synthesis

Across pathogen systems, *Apodemus* spp. occupy heterogeneous epidemiological roles rather than a uniform reservoir status. Their contribution spans a continuum from confirmed reservoir competence to context-dependent involvement or incidental participation within broader transmission networks.

Strong evidence supports a central reservoir role for *Borrelia* spp., *Neoehrlichia mikurensis*, and certain *Babesia* spp., in which sustained rodent–tick cycles underpin local enzootic maintenance. In contrast, the role of *Anaplasma phagocytophilum* is ecotype-structured and geographically variable, reflecting differences in host associations and community composition. For *Rickettsia* spp. and *Ehrlichia muris*, pathogen detection in *Apodemus* is documented, yet definitive reservoir competence remains uncertain. At the opposite end of the gradient, *Coxiella burnetii*, flaviviruses, and Crimean–Congo haemorrhagic fever virus are maintained primarily by alternative hosts or transmission pathways, with rodents playing limited or indirect roles.

**Figure 1:**
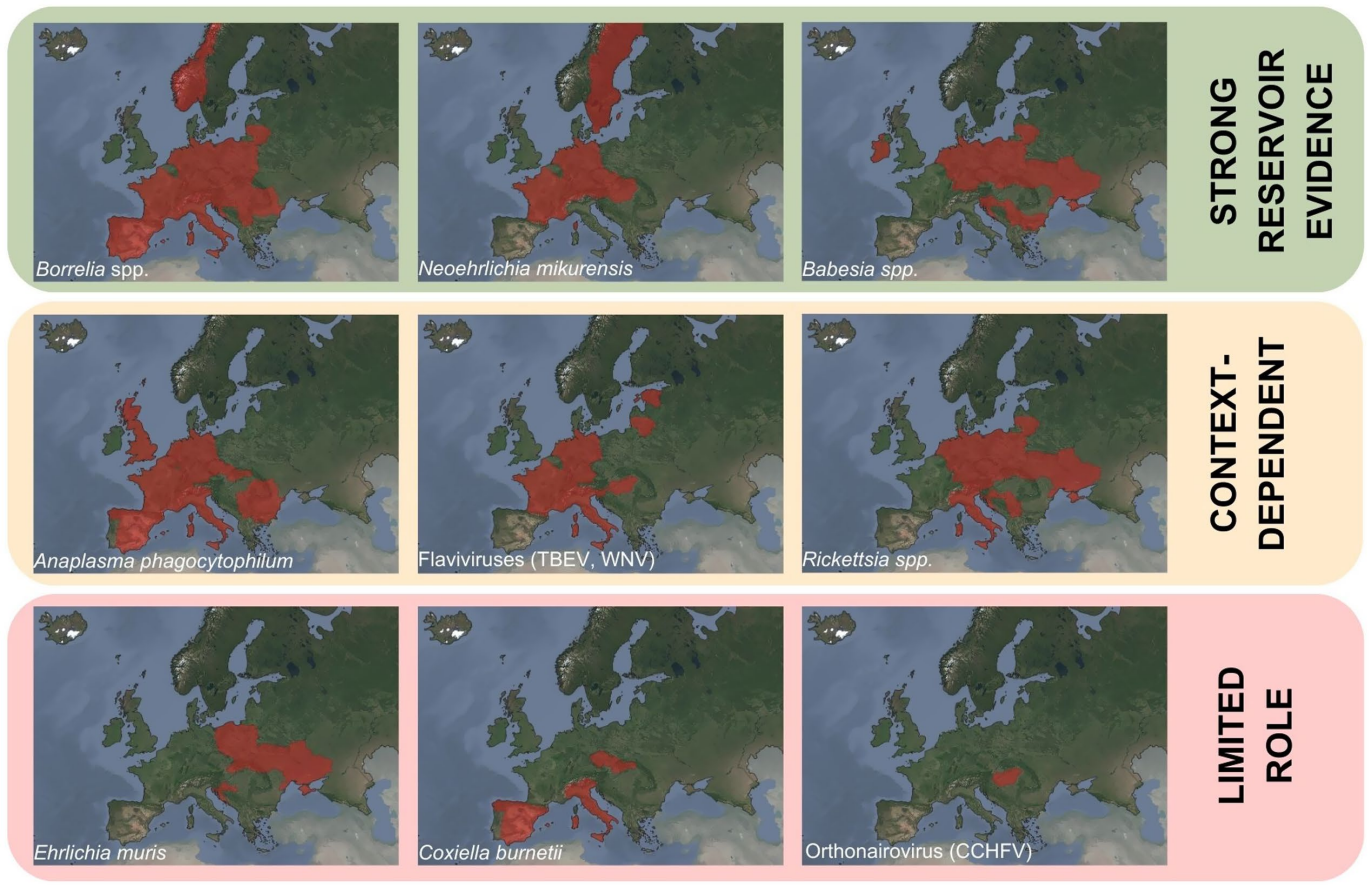
Spatial distribution of reported vector-borne pathogen detections in *Apodemus* spp. Panels are arranged according to inferred epidemiological role, ranging from confirmed reservoir associations to incidental or limited involvement. Countries shaded in red indicate the presence of published detections and do not imply complete or homogeneous pathogen distribution within national borders.

These contrasts are mirrored in the spatial distribution of reported detections across Europe (Figure 1), which highlights broad reporting for confirmed rodent-associated pathogens and more fragmented patterns for context-dependent or incidental agents. Importantly, detection patterns reflect both ecological processes and variation in sampling intensity.

Overall, *Apodemus* spp. function as context-dependent nodes within multi-host, multi-vector systems, and their epidemiological relevance emerges from interactions among host demography, vector biology, and community structure rather than from uniform reservoir status.

## Implications for One Health surveillance and future research

The reviewed evidence supports a One Health interpretation in which zoonotic risk emerges from the interaction of rodent ecology, vector biology, habitat structure, and environmental change. *Apodemus* spp. occur across forested, agricultural, and peri-urban landscapes and frequently host immature vector stages. As a result, their responses to climate and land-use change can influence both enzootic maintenance and patterns of human exposure (75, 105, 107, 111). Landscape structure, forest cover, and temperature-related variables therefore link *Apodemus* ecology directly to large-scale variation in tick-borne disease risk (104, 112, 113, 117, 118).

Future research should prioritise evidence that moves beyond host detection alone. The most informative studies will integrate rodent infection data with vector burdens, vector infection prevalence, genotype- or ecotype-resolved pathogen identification, and direct tests of host competence (31, 56, 57). Longitudinal designs are particularly important, because host density, tick burden, and infected nymph density are often decoupled through time (76, 93, 94). More explicit comparison among *Apodemus* species, especially where morphological identification is difficult, would also help clarify whether epidemiological roles differ consistently among *A. flavicollis*, *A. sylvaticus*, *A. agrarius*, and less frequently studied taxa (14). Finally, broader surveillance in underrepresented regions and pathogen systems is needed to reduce the current geographic and taxonomic bias of the evidence base (34, 75, 108).

## Strengths and limitations of the review

A major strength of this review is its comparative perspective across viral, bacterial, and protozoan pathogen systems. This makes it possible to distinguish pathogens for which *Apodemus* hosts are strongly implicated from those for which evidence remains limited, indirect, or ecotype-dependent, as shown by the contrast between *Borrelia* spp. and *Neoehrlichia mikurensis* on one hand, and WNV, CCHFV, and *Coxiella burnetii* on the other (30, 32, 38, 46, 79, 81, 119). It also allows host role to be interpreted in the context of vector biology, habitat structure, and pathogen life history rather than from prevalence data alone (63, 75, 104).

Several limitations should also be recognised. First, this is a narrative synthesis informed by structured literature retrieval rather than a formal systematic review or meta-analysis. Second, the included studies were highly heterogeneous in geography, sampling design, host identification, sample matrix, and diagnostic method. PCR, serology, blood smears, and microscopical analyses are not directly comparable, and positive detections in tissues or sera do not in themselves demonstrate infectivity to vectors or sustained transmission competence (31, 34, 35, 55). Third, species-level host identification was not always available, and some studies reported only *Apodemus* spp., which may obscure species-specific patterns, an issue that is particularly relevant given the morphological similarity among European taxa (14). Fourth, the evidence base was uneven across pathogens, with dense literature for tick-borne bacteria such as *Borrelia* spp. but sparse evidence for WNV or CCHFV (38, 79, 109, 120). Finally, geographic reporting intensity varied across Europe, so apparent absence in some regions may reflect surveillance effort rather than true ecological absence (34, 75).
