# Supplementary material for: From reservoirs to ecological integrators: the role of European Apodemus spp. in vector-borne zoonotic pathogens
Source: Front Vet Sci. 2026 Jun 4;13:1822333. doi: 10.3389/fvets.2026.1822333 (PMC13275226; doi:10.3389/fvets.2026.1822333)
Supplement: Supplementary file 1 [file Table_1.DOCX]

**Supplementary Material**

*Table S1. Database-specific search queries used for structured literature retrieval*

Search strings are reported as executed or as database-adapted equivalents of the core conceptual query; syntax varied among databases to accommodate platform-specific indexing and search operators

**PubMed**

| **Database** | **Pathogen / topic** | **Search query** |
| --- | --- | --- |
| PubMed | General capture query | ("Apodemus OR "wood mouse" OR "yellow-necked mouse" OR "striped field mouse""[Title/Abstract]) AND (tick*[Title/Abstract] OR flea*[Title/Abstract] OR mite*[Title/Abstract] OR vector-borne[Title/Abstract]) AND (Europe OR Austria OR Belarus OR Belgium OR Bosnia OR Bulgaria OR Croatia OR Czechia OR "Czech Republic" OR Denmark OR Estonia OR Finland OR France OR Germany OR Greece OR Hungary OR Ireland OR Italy OR Latvia OR Lithuania OR Luxembourg OR Moldova OR Montenegro OR Netherlands OR Norway OR Poland OR Portugal OR Romania OR Serbia OR Slovakia OR Slovenia OR Spain OR Sweden OR Switzerland OR Ukraine OR "United Kingdom" OR England OR Scotland OR Wales[Title/Abstract]) |
| PubMed | TBEV | ("Apodemus OR "wood mouse" OR "yellow-necked mouse" OR "striped field mouse""[Title/Abstract]) AND ("tick-borne encephalitis virus"[Title/Abstract] OR TBEV[Title/Abstract] OR "Orthoflavivirus encephalitidis"[Title/Abstract]) AND (Europe OR Austria OR Belarus OR Belgium OR Bosnia OR Bulgaria OR Croatia OR Czechia OR "Czech Republic" OR Denmark OR Estonia OR Finland OR France OR Germany OR Greece OR Hungary OR Ireland OR Italy OR Latvia OR Lithuania OR Luxembourg OR Moldova OR Montenegro OR Netherlands OR Norway OR Poland OR Portugal OR Romania OR Serbia OR Slovakia OR Slovenia OR Spain OR Sweden OR Switzerland OR Ukraine OR "United Kingdom" OR England OR Scotland OR Wales[Title/Abstract]) |
| PubMed | West Nile virus | ("Apodemus OR "wood mouse" OR "yellow-necked mouse" OR "striped field mouse""[Title/Abstract]) AND ("West Nile virus"[Title/Abstract] OR WNV[Title/Abstract] OR "Orthoflavivirus nilense"[Title/Abstract]) AND (Europe OR Austria OR Belarus OR Belgium OR Bosnia OR Bulgaria OR Croatia OR Czechia OR "Czech Republic" OR Denmark OR Estonia OR Finland OR France OR Germany OR Greece OR Hungary OR Ireland OR Italy OR Latvia OR Lithuania OR Luxembourg OR Moldova OR Montenegro OR Netherlands OR Norway OR Poland OR Portugal OR Romania OR Serbia OR Slovakia OR Slovenia OR Spain OR Sweden OR Switzerland OR Ukraine OR "United Kingdom" OR England OR Scotland OR Wales[Title/Abstract]) |
| PubMed | Borrelia burgdorferi s.l. / B. miyamotoi | ("Apodemus OR "wood mouse" OR "yellow-necked mouse" OR "striped field mouse""[Title/Abstract]) AND ("Borrelia burgdorferi"[Title/Abstract] OR "Borrelia burgdorferi sensu lato"[Title/Abstract] OR "Borrelia afzelii"[Title/Abstract] OR "Borrelia garinii"[Title/Abstract] OR "Borrelia miyamotoi"[Title/Abstract] OR Lyme[Title/Abstract]) AND (Europe OR Austria OR Belarus OR Belgium OR Bosnia OR Bulgaria OR Croatia OR Czechia OR "Czech Republic" OR Denmark OR Estonia OR Finland OR France OR Germany OR Greece OR Hungary OR Ireland OR Italy OR Latvia OR Lithuania OR Luxembourg OR Moldova OR Montenegro OR Netherlands OR Norway OR Poland OR Portugal OR Romania OR Serbia OR Slovakia OR Slovenia OR Spain OR Sweden OR Switzerland OR Ukraine OR "United Kingdom" OR England OR Scotland OR Wales[Title/Abstract]) |
| PubMed | Ehrlichia muris | ("Apodemus OR "wood mouse" OR "yellow-necked mouse" OR "striped field mouse""[Title/Abstract]) AND ("Ehrlichia muris"[Title/Abstract] OR "Ehrlichia muris-like"[Title/Abstract]) AND (Europe OR Austria OR Belarus OR Belgium OR Bosnia OR Bulgaria OR Croatia OR Czechia OR "Czech Republic" OR Denmark OR Estonia OR Finland OR France OR Germany OR Greece OR Hungary OR Ireland OR Italy OR Latvia OR Lithuania OR Luxembourg OR Moldova OR Montenegro OR Netherlands OR Norway OR Poland OR Portugal OR Romania OR Serbia OR Slovakia OR Slovenia OR Spain OR Sweden OR Switzerland OR Ukraine OR "United Kingdom" OR England OR Scotland OR Wales[Title/Abstract]) |
| PubMed | Neoehrlichia mikurensis | ("Apodemus OR "wood mouse" OR "yellow-necked mouse" OR "striped field mouse""[Title/Abstract]) AND ("Neoehrlichia mikurensis"[Title/Abstract] OR "Candidatus Neoehrlichia mikurensis"[Title/Abstract]) AND (Europe OR Austria OR Belarus OR Belgium OR Bosnia OR Bulgaria OR Croatia OR Czechia OR "Czech Republic" OR Denmark OR Estonia OR Finland OR France OR Germany OR Greece OR Hungary OR Ireland OR Italy OR Latvia OR Lithuania OR Luxembourg OR Moldova OR Montenegro OR Netherlands OR Norway OR Poland OR Portugal OR Romania OR Serbia OR Slovakia OR Slovenia OR Spain OR Sweden OR Switzerland OR Ukraine OR "United Kingdom" OR England OR Scotland OR Wales[Title/Abstract]) |
| PubMed | Rickettsia spp. | ("Apodemus OR "wood mouse" OR "yellow-necked mouse" OR "striped field mouse""[Title/Abstract]) AND (Rickettsia[Title/Abstract] OR "Rickettsia helvetica"[Title/Abstract] OR "Rickettsia slovaca"[Title/Abstract] OR "Rickettsia conorii"[Title/Abstract] OR "Rickettsia felis"[Title/Abstract]) AND (Europe OR Austria OR Belarus OR Belgium OR Bosnia OR Bulgaria OR Croatia OR Czechia OR "Czech Republic" OR Denmark OR Estonia OR Finland OR France OR Germany OR Greece OR Hungary OR Ireland OR Italy OR Latvia OR Lithuania OR Luxembourg OR Moldova OR Montenegro OR Netherlands OR Norway OR Poland OR Portugal OR Romania OR Serbia OR Slovakia OR Slovenia OR Spain OR Sweden OR Switzerland OR Ukraine OR "United Kingdom" OR England OR Scotland OR Wales[Title/Abstract]) |
| PubMed | Babesia spp. | ("Apodemus OR "wood mouse" OR "yellow-necked mouse" OR "striped field mouse""[Title/Abstract]) AND (Babesia[Title/Abstract] OR "Babesia microti"[Title/Abstract]) AND (Europe OR Austria OR Belarus OR Belgium OR Bosnia OR Bulgaria OR Croatia OR Czechia OR "Czech Republic" OR Denmark OR Estonia OR Finland OR France OR Germany OR Greece OR Hungary OR Ireland OR Italy OR Latvia OR Lithuania OR Luxembourg OR Moldova OR Montenegro OR Netherlands OR Norway OR Poland OR Portugal OR Romania OR Serbia OR Slovakia OR Slovenia OR Spain OR Sweden OR Switzerland OR Ukraine OR "United Kingdom" OR England OR Scotland OR Wales[Title/Abstract]) |
| PubMed | Crimean-Congo haemorrhagic fever virus | ("Apodemus OR "wood mouse" OR "yellow-necked mouse" OR "striped field mouse""[Title/Abstract]) AND ("Crimean-Congo hemorrhagic fever virus"[Title/Abstract] OR "Crimean-Congo haemorrhagic fever virus"[Title/Abstract] OR CCHFV[Title/Abstract]) AND (Europe OR Austria OR Belarus OR Belgium OR Bosnia OR Bulgaria OR Croatia OR Czechia OR "Czech Republic" OR Denmark OR Estonia OR Finland OR France OR Germany OR Greece OR Hungary OR Ireland OR Italy OR Latvia OR Lithuania OR Luxembourg OR Moldova OR Montenegro OR Netherlands OR Norway OR Poland OR Portugal OR Romania OR Serbia OR Slovakia OR Slovenia OR Spain OR Sweden OR Switzerland OR Ukraine OR "United Kingdom" OR England OR Scotland OR Wales[Title/Abstract]) |
| PubMed | Anaplasma phagocytophilum | ("Apodemus OR "wood mouse" OR "yellow-necked mouse" OR "striped field mouse""[Title/Abstract]) AND ("Anaplasma phagocytophilum"[Title/Abstract] OR anaplasmosis[Title/Abstract]) AND (Europe OR Austria OR Belarus OR Belgium OR Bosnia OR Bulgaria OR Croatia OR Czechia OR "Czech Republic" OR Denmark OR Estonia OR Finland OR France OR Germany OR Greece OR Hungary OR Ireland OR Italy OR Latvia OR Lithuania OR Luxembourg OR Moldova OR Montenegro OR Netherlands OR Norway OR Poland OR Portugal OR Romania OR Serbia OR Slovakia OR Slovenia OR Spain OR Sweden OR Switzerland OR Ukraine OR "United Kingdom" OR England OR Scotland OR Wales[Title/Abstract]) |
| PubMed | Coxiella burnetii | ("Apodemus OR "wood mouse" OR "yellow-necked mouse" OR "striped field mouse""[Title/Abstract]) AND ("Coxiella burnetii"[Title/Abstract] OR "Q fever"[Title/Abstract]) AND (Europe OR Austria OR Belarus OR Belgium OR Bosnia OR Bulgaria OR Croatia OR Czechia OR "Czech Republic" OR Denmark OR Estonia OR Finland OR France OR Germany OR Greece OR Hungary OR Ireland OR Italy OR Latvia OR Lithuania OR Luxembourg OR Moldova OR Montenegro OR Netherlands OR Norway OR Poland OR Portugal OR Romania OR Serbia OR Slovakia OR Slovenia OR Spain OR Sweden OR Switzerland OR Ukraine OR "United Kingdom" OR England OR Scotland OR Wales[Title/Abstract]) |

**Web of Science**

| **Database** | **Pathogen / topic** | **Search query** |
| --- | --- | --- |
| Web of Science | General capture query | TS=((Apodemus OR "wood mouse" OR "yellow-necked mouse" OR "striped field mouse") AND (tick* OR flea* OR mite* OR "vector-borne") AND (Europe OR Austria OR Belarus OR Belgium OR Bosnia OR Bulgaria OR Croatia OR Czechia OR "Czech Republic" OR Denmark OR Estonia OR Finland OR France OR Germany OR Greece OR Hungary OR Ireland OR Italy OR Latvia OR Lithuania OR Luxembourg OR Moldova OR Montenegro OR Netherlands OR Norway OR Poland OR Portugal OR Romania OR Serbia OR Slovakia OR Slovenia OR Spain OR Sweden OR Switzerland OR Ukraine OR "United Kingdom" OR England OR Scotland OR Wales)) |
| Web of Science | TBEV | TS=((Apodemus OR "wood mouse" OR "yellow-necked mouse" OR "striped field mouse") AND ("tick-borne encephalitis virus" OR TBEV OR "Orthoflavivirus encephalitidis") AND (Europe OR Austria OR Belarus OR Belgium OR Bosnia OR Bulgaria OR Croatia OR Czechia OR "Czech Republic" OR Denmark OR Estonia OR Finland OR France OR Germany OR Greece OR Hungary OR Ireland OR Italy OR Latvia OR Lithuania OR Luxembourg OR Moldova OR Montenegro OR Netherlands OR Norway OR Poland OR Portugal OR Romania OR Serbia OR Slovakia OR Slovenia OR Spain OR Sweden OR Switzerland OR Ukraine OR "United Kingdom" OR England OR Scotland OR Wales)) |
| Web of Science | West Nile virus | TS=((Apodemus OR "wood mouse" OR "yellow-necked mouse" OR "striped field mouse") AND ("West Nile virus" OR WNV OR "Orthoflavivirus nilense") AND (Europe OR Austria OR Belarus OR Belgium OR Bosnia OR Bulgaria OR Croatia OR Czechia OR "Czech Republic" OR Denmark OR Estonia OR Finland OR France OR Germany OR Greece OR Hungary OR Ireland OR Italy OR Latvia OR Lithuania OR Luxembourg OR Moldova OR Montenegro OR Netherlands OR Norway OR Poland OR Portugal OR Romania OR Serbia OR Slovakia OR Slovenia OR Spain OR Sweden OR Switzerland OR Ukraine OR "United Kingdom" OR England OR Scotland OR Wales)) |
| Web of Science | Borrelia burgdorferi s.l. / B. miyamotoi | TS=((Apodemus OR "wood mouse" OR "yellow-necked mouse" OR "striped field mouse") AND ("Borrelia burgdorferi" OR "Borrelia burgdorferi sensu lato" OR "Borrelia afzelii" OR "Borrelia garinii" OR "Borrelia miyamotoi" OR Lyme) AND (Europe OR Austria OR Belarus OR Belgium OR Bosnia OR Bulgaria OR Croatia OR Czechia OR "Czech Republic" OR Denmark OR Estonia OR Finland OR France OR Germany OR Greece OR Hungary OR Ireland OR Italy OR Latvia OR Lithuania OR Luxembourg OR Moldova OR Montenegro OR Netherlands OR Norway OR Poland OR Portugal OR Romania OR Serbia OR Slovakia OR Slovenia OR Spain OR Sweden OR Switzerland OR Ukraine OR "United Kingdom" OR England OR Scotland OR Wales)) |
| Web of Science | Ehrlichia muris | TS=((Apodemus OR "wood mouse" OR "yellow-necked mouse" OR "striped field mouse") AND ("Ehrlichia muris" OR "Ehrlichia muris-like") AND (Europe OR Austria OR Belarus OR Belgium OR Bosnia OR Bulgaria OR Croatia OR Czechia OR "Czech Republic" OR Denmark OR Estonia OR Finland OR France OR Germany OR Greece OR Hungary OR Ireland OR Italy OR Latvia OR Lithuania OR Luxembourg OR Moldova OR Montenegro OR Netherlands OR Norway OR Poland OR Portugal OR Romania OR Serbia OR Slovakia OR Slovenia OR Spain OR Sweden OR Switzerland OR Ukraine OR "United Kingdom" OR England OR Scotland OR Wales)) |
| Web of Science | Neoehrlichia mikurensis | TS=((Apodemus OR "wood mouse" OR "yellow-necked mouse" OR "striped field mouse") AND ("Neoehrlichia mikurensis" OR "Candidatus Neoehrlichia mikurensis") AND (Europe OR Austria OR Belarus OR Belgium OR Bosnia OR Bulgaria OR Croatia OR Czechia OR "Czech Republic" OR Denmark OR Estonia OR Finland OR France OR Germany OR Greece OR Hungary OR Ireland OR Italy OR Latvia OR Lithuania OR Luxembourg OR Moldova OR Montenegro OR Netherlands OR Norway OR Poland OR Portugal OR Romania OR Serbia OR Slovakia OR Slovenia OR Spain OR Sweden OR Switzerland OR Ukraine OR "United Kingdom" OR England OR Scotland OR Wales)) |
| Web of Science | Rickettsia spp. | TS=((Apodemus OR "wood mouse" OR "yellow-necked mouse" OR "striped field mouse") AND (Rickettsia OR "Rickettsia helvetica" OR "Rickettsia slovaca" OR "Rickettsia conorii" OR "Rickettsia felis") AND (Europe OR Austria OR Belarus OR Belgium OR Bosnia OR Bulgaria OR Croatia OR Czechia OR "Czech Republic" OR Denmark OR Estonia OR Finland OR France OR Germany OR Greece OR Hungary OR Ireland OR Italy OR Latvia OR Lithuania OR Luxembourg OR Moldova OR Montenegro OR Netherlands OR Norway OR Poland OR Portugal OR Romania OR Serbia OR Slovakia OR Slovenia OR Spain OR Sweden OR Switzerland OR Ukraine OR "United Kingdom" OR England OR Scotland OR Wales)) |
| Web of Science | Babesia spp. | TS=((Apodemus OR "wood mouse" OR "yellow-necked mouse" OR "striped field mouse") AND (Babesia OR "Babesia microti") AND (Europe OR Austria OR Belarus OR Belgium OR Bosnia OR Bulgaria OR Croatia OR Czechia OR "Czech Republic" OR Denmark OR Estonia OR Finland OR France OR Germany OR Greece OR Hungary OR Ireland OR Italy OR Latvia OR Lithuania OR Luxembourg OR Moldova OR Montenegro OR Netherlands OR Norway OR Poland OR Portugal OR Romania OR Serbia OR Slovakia OR Slovenia OR Spain OR Sweden OR Switzerland OR Ukraine OR "United Kingdom" OR England OR Scotland OR Wales)) |
| Web of Science | Crimean-Congo haemorrhagic fever virus | TS=((Apodemus OR "wood mouse" OR "yellow-necked mouse" OR "striped field mouse") AND ("Crimean-Congo hemorrhagic fever virus" OR "Crimean-Congo haemorrhagic fever virus" OR CCHFV) AND (Europe OR Austria OR Belarus OR Belgium OR Bosnia OR Bulgaria OR Croatia OR Czechia OR "Czech Republic" OR Denmark OR Estonia OR Finland OR France OR Germany OR Greece OR Hungary OR Ireland OR Italy OR Latvia OR Lithuania OR Luxembourg OR Moldova OR Montenegro OR Netherlands OR Norway OR Poland OR Portugal OR Romania OR Serbia OR Slovakia OR Slovenia OR Spain OR Sweden OR Switzerland OR Ukraine OR "United Kingdom" OR England OR Scotland OR Wales)) |
| Web of Science | Anaplasma phagocytophilum | TS=((Apodemus OR "wood mouse" OR "yellow-necked mouse" OR "striped field mouse") AND ("Anaplasma phagocytophilum" OR anaplasmosis) AND (Europe OR Austria OR Belarus OR Belgium OR Bosnia OR Bulgaria OR Croatia OR Czechia OR "Czech Republic" OR Denmark OR Estonia OR Finland OR France OR Germany OR Greece OR Hungary OR Ireland OR Italy OR Latvia OR Lithuania OR Luxembourg OR Moldova OR Montenegro OR Netherlands OR Norway OR Poland OR Portugal OR Romania OR Serbia OR Slovakia OR Slovenia OR Spain OR Sweden OR Switzerland OR Ukraine OR "United Kingdom" OR England OR Scotland OR Wales)) |
| Web of Science | Coxiella burnetii | TS=((Apodemus OR "wood mouse" OR "yellow-necked mouse" OR "striped field mouse") AND ("Coxiella burnetii" OR "Q fever") AND (Europe OR Austria OR Belarus OR Belgium OR Bosnia OR Bulgaria OR Croatia OR Czechia OR "Czech Republic" OR Denmark OR Estonia OR Finland OR France OR Germany OR Greece OR Hungary OR Ireland OR Italy OR Latvia OR Lithuania OR Luxembourg OR Moldova OR Montenegro OR Netherlands OR Norway OR Poland OR Portugal OR Romania OR Serbia OR Slovakia OR Slovenia OR Spain OR Sweden OR Switzerland OR Ukraine OR "United Kingdom" OR England OR Scotland OR Wales)) |

**Google Scholar**

| **Database** | **Pathogen / topic** | **Search query** |
| --- | --- | --- |
| Google Scholar | General capture query | "Apodemus" tick OR flea OR mite OR "vector-borne" Europe |
| Google Scholar | TBEV | "Apodemus" "tick-borne encephalitis virus" OR TBEV Europe |
| Google Scholar | West Nile virus | "Apodemus" "West Nile virus" OR WNV Europe |
| Google Scholar | Borrelia burgdorferi s.l. / B. miyamotoi | "Apodemus" "Borrelia burgdorferi" OR "Borrelia afzelii" OR "Borrelia miyamotoi" Europe |
| Google Scholar | Ehrlichia muris | "Apodemus" "Ehrlichia muris" Europe |
| Google Scholar | Neoehrlichia mikurensis | "Apodemus" "Neoehrlichia mikurensis" OR "Candidatus Neoehrlichia mikurensis" Europe |
| Google Scholar | Rickettsia spp. | "Apodemus" Rickettsia OR "Rickettsia helvetica" OR "Rickettsia slovaca" Europe |
| Google Scholar | Babesia spp. | "Apodemus" Babesia OR "Babesia microti" Europe |
| Google Scholar | Crimean-Congo haemorrhagic fever virus | "Apodemus" "Crimean-Congo hemorrhagic fever virus" OR CCHFV Europe |
| Google Scholar | Anaplasma phagocytophilum | "Apodemus" "Anaplasma phagocytophilum" Europe |
| Google Scholar | Coxiella burnetii | "Apodemus" "Coxiella burnetii" OR "Q fever" Europe |

**CABI Digital Library**

| **Database** | **Pathogen / topic** | **Search query** |
| --- | --- | --- |
| CABI Digital Library | General capture query | (Apodemus OR "wood mouse" OR "yellow-necked mouse" OR "striped field mouse") AND (tick* OR flea* OR mite* OR "vector-borne") AND (Europe OR Austria OR Belarus OR Belgium OR Bosnia OR Bulgaria OR Croatia OR Czechia OR "Czech Republic" OR Denmark OR Estonia OR Finland OR France OR Germany OR Greece OR Hungary OR Ireland OR Italy OR Latvia OR Lithuania OR Luxembourg OR Moldova OR Montenegro OR Netherlands OR Norway OR Poland OR Portugal OR Romania OR Serbia OR Slovakia OR Slovenia OR Spain OR Sweden OR Switzerland OR Ukraine OR "United Kingdom" OR England OR Scotland OR Wales) |
| CABI Digital Library | TBEV | (Apodemus OR "wood mouse" OR "yellow-necked mouse" OR "striped field mouse") AND ("tick-borne encephalitis virus" OR TBEV OR "Orthoflavivirus encephalitidis") AND (Europe OR Austria OR Belarus OR Belgium OR Bosnia OR Bulgaria OR Croatia OR Czechia OR "Czech Republic" OR Denmark OR Estonia OR Finland OR France OR Germany OR Greece OR Hungary OR Ireland OR Italy OR Latvia OR Lithuania OR Luxembourg OR Moldova OR Montenegro OR Netherlands OR Norway OR Poland OR Portugal OR Romania OR Serbia OR Slovakia OR Slovenia OR Spain OR Sweden OR Switzerland OR Ukraine OR "United Kingdom" OR England OR Scotland OR Wales) |
| CABI Digital Library | West Nile virus | (Apodemus OR "wood mouse" OR "yellow-necked mouse" OR "striped field mouse") AND ("West Nile virus" OR WNV OR "Orthoflavivirus nilense") AND (Europe OR Austria OR Belarus OR Belgium OR Bosnia OR Bulgaria OR Croatia OR Czechia OR "Czech Republic" OR Denmark OR Estonia OR Finland OR France OR Germany OR Greece OR Hungary OR Ireland OR Italy OR Latvia OR Lithuania OR Luxembourg OR Moldova OR Montenegro OR Netherlands OR Norway OR Poland OR Portugal OR Romania OR Serbia OR Slovakia OR Slovenia OR Spain OR Sweden OR Switzerland OR Ukraine OR "United Kingdom" OR England OR Scotland OR Wales) |
| CABI Digital Library | Borrelia burgdorferi s.l. / B. miyamotoi | (Apodemus OR "wood mouse" OR "yellow-necked mouse" OR "striped field mouse") AND ("Borrelia burgdorferi" OR "Borrelia burgdorferi sensu lato" OR "Borrelia afzelii" OR "Borrelia garinii" OR "Borrelia miyamotoi" OR Lyme) AND (Europe OR Austria OR Belarus OR Belgium OR Bosnia OR Bulgaria OR Croatia OR Czechia OR "Czech Republic" OR Denmark OR Estonia OR Finland OR France OR Germany OR Greece OR Hungary OR Ireland OR Italy OR Latvia OR Lithuania OR Luxembourg OR Moldova OR Montenegro OR Netherlands OR Norway OR Poland OR Portugal OR Romania OR Serbia OR Slovakia OR Slovenia OR Spain OR Sweden OR Switzerland OR Ukraine OR "United Kingdom" OR England OR Scotland OR Wales) |
| CABI Digital Library | Ehrlichia muris | (Apodemus OR "wood mouse" OR "yellow-necked mouse" OR "striped field mouse") AND ("Ehrlichia muris" OR "Ehrlichia muris-like") AND (Europe OR Austria OR Belarus OR Belgium OR Bosnia OR Bulgaria OR Croatia OR Czechia OR "Czech Republic" OR Denmark OR Estonia OR Finland OR France OR Germany OR Greece OR Hungary OR Ireland OR Italy OR Latvia OR Lithuania OR Luxembourg OR Moldova OR Montenegro OR Netherlands OR Norway OR Poland OR Portugal OR Romania OR Serbia OR Slovakia OR Slovenia OR Spain OR Sweden OR Switzerland OR Ukraine OR "United Kingdom" OR England OR Scotland OR Wales) |
| CABI Digital Library | Neoehrlichia mikurensis | (Apodemus OR "wood mouse" OR "yellow-necked mouse" OR "striped field mouse") AND ("Neoehrlichia mikurensis" OR "Candidatus Neoehrlichia mikurensis") AND (Europe OR Austria OR Belarus OR Belgium OR Bosnia OR Bulgaria OR Croatia OR Czechia OR "Czech Republic" OR Denmark OR Estonia OR Finland OR France OR Germany OR Greece OR Hungary OR Ireland OR Italy OR Latvia OR Lithuania OR Luxembourg OR Moldova OR Montenegro OR Netherlands OR Norway OR Poland OR Portugal OR Romania OR Serbia OR Slovakia OR Slovenia OR Spain OR Sweden OR Switzerland OR Ukraine OR "United Kingdom" OR England OR Scotland OR Wales) |
| CABI Digital Library | Rickettsia spp. | (Apodemus OR "wood mouse" OR "yellow-necked mouse" OR "striped field mouse") AND (Rickettsia OR "Rickettsia helvetica" OR "Rickettsia slovaca" OR "Rickettsia conorii" OR "Rickettsia felis") AND (Europe OR Austria OR Belarus OR Belgium OR Bosnia OR Bulgaria OR Croatia OR Czechia OR "Czech Republic" OR Denmark OR Estonia OR Finland OR France OR Germany OR Greece OR Hungary OR Ireland OR Italy OR Latvia OR Lithuania OR Luxembourg OR Moldova OR Montenegro OR Netherlands OR Norway OR Poland OR Portugal OR Romania OR Serbia OR Slovakia OR Slovenia OR Spain OR Sweden OR Switzerland OR Ukraine OR "United Kingdom" OR England OR Scotland OR Wales) |
| CABI Digital Library | Babesia spp. | (Apodemus OR "wood mouse" OR "yellow-necked mouse" OR "striped field mouse") AND (Babesia OR "Babesia microti") AND (Europe OR Austria OR Belarus OR Belgium OR Bosnia OR Bulgaria OR Croatia OR Czechia OR "Czech Republic" OR Denmark OR Estonia OR Finland OR France OR Germany OR Greece OR Hungary OR Ireland OR Italy OR Latvia OR Lithuania OR Luxembourg OR Moldova OR Montenegro OR Netherlands OR Norway OR Poland OR Portugal OR Romania OR Serbia OR Slovakia OR Slovenia OR Spain OR Sweden OR Switzerland OR Ukraine OR "United Kingdom" OR England OR Scotland OR Wales) |
| CABI Digital Library | Crimean-Congo haemorrhagic fever virus | (Apodemus OR "wood mouse" OR "yellow-necked mouse" OR "striped field mouse") AND ("Crimean-Congo hemorrhagic fever virus" OR "Crimean-Congo haemorrhagic fever virus" OR CCHFV) AND (Europe OR Austria OR Belarus OR Belgium OR Bosnia OR Bulgaria OR Croatia OR Czechia OR "Czech Republic" OR Denmark OR Estonia OR Finland OR France OR Germany OR Greece OR Hungary OR Ireland OR Italy OR Latvia OR Lithuania OR Luxembourg OR Moldova OR Montenegro OR Netherlands OR Norway OR Poland OR Portugal OR Romania OR Serbia OR Slovakia OR Slovenia OR Spain OR Sweden OR Switzerland OR Ukraine OR "United Kingdom" OR England OR Scotland OR Wales) |
| CABI Digital Library | Anaplasma phagocytophilum | (Apodemus OR "wood mouse" OR "yellow-necked mouse" OR "striped field mouse") AND ("Anaplasma phagocytophilum" OR anaplasmosis) AND (Europe OR Austria OR Belarus OR Belgium OR Bosnia OR Bulgaria OR Croatia OR Czechia OR "Czech Republic" OR Denmark OR Estonia OR Finland OR France OR Germany OR Greece OR Hungary OR Ireland OR Italy OR Latvia OR Lithuania OR Luxembourg OR Moldova OR Montenegro OR Netherlands OR Norway OR Poland OR Portugal OR Romania OR Serbia OR Slovakia OR Slovenia OR Spain OR Sweden OR Switzerland OR Ukraine OR "United Kingdom" OR England OR Scotland OR Wales) |
| CABI Digital Library | Coxiella burnetii | (Apodemus OR "wood mouse" OR "yellow-necked mouse" OR "striped field mouse") AND ("Coxiella burnetii" OR "Q fever") AND (Europe OR Austria OR Belarus OR Belgium OR Bosnia OR Bulgaria OR Croatia OR Czechia OR "Czech Republic" OR Denmark OR Estonia OR Finland OR France OR Germany OR Greece OR Hungary OR Ireland OR Italy OR Latvia OR Lithuania OR Luxembourg OR Moldova OR Montenegro OR Netherlands OR Norway OR Poland OR Portugal OR Romania OR Serbia OR Slovakia OR Slovenia OR Spain OR Sweden OR Switzerland OR Ukraine OR "United Kingdom" OR England OR Scotland OR Wales) |
